# Development and psychometric evaluation of the Job Demands in Nursing Scale and Job Resources in Nursing Scale: Results from a national study

**DOI:** 10.1002/nop2.215

**Published:** 2018-11-13

**Authors:** Kelly L. Penz, Julie G. Kosteniuk, Norma J. Stewart, Martha L. P. MacLeod, Judith C. Kulig, Chandima P. Karunanayake, Kelley Kilpatrick

**Affiliations:** ^1^ College of Nursing University of Saskatchewan Regina Saskatchewan Canada; ^2^ Canadian Centre for Health and Safety in Agriculture, College of Medicine University of Saskatchewan Saskatoon Saskatchewan Canada; ^3^ College of Nursing University of Saskatchewan Saskatoon Saskatchewan Canada; ^4^ School of Nursing University of Northern British Columbia Prince George British Columbia Canada; ^5^ Faculty of Health Sciences University of Lethbridge Lethbridge Alberta Canada; ^6^ Canadian Centre for Health and Safety in Agriculture University of Saskatchewan Saskatoon Saskatchewan Canada; ^7^ Faculty of Nursing, CIUSSS EIM‐Maisonneuve‐Rosemont Hospital Site Université de Montréal Montréal Québec Canada

**Keywords:** burnout, cross‐sectional survey, instrument development, job demands, job resources, nursing, work engagement

## Abstract

**Aim:**

To develop and test the psychometric properties of the Job Resources in Nursing (JRIN) Scale and the Job Demands in Nursing (JDIN) Scale.

**Design:**

Cross‐sectional survey.

**Methods:**

A three‐phase process of instrument development and psychometric evaluation was employed: Phase 1: development of a 42‐item JRIN Scale and 60‐item JDIN Scale through extensive literature review, expert consultation and an iterative content evaluation; Phase 2: pilot survey of 89 nurses and use of item discrimination analysis to estimate the internal consistency reliability of each subscale and reduce the length of each scale; Phase 3: Modified scales were tested in a nationwide survey of 3,822 rural/remote nurses, including use of exploratory factor analysis.

**Results:**

The 24 items related to job resources favoured a six‐factor structure, accounting for 63% of the variance, Cronbach's alpha 0.88. The 22 items related to job demands favoured a six‐factor structure, accounting for 59% of the variance, Cronbach's alpha 0.84.

## 1 INTRODUCTION

Studies have shown that specific occupational circumstances have measurable effects on nurses’ professional well‐being. Work stress is a major concern in the nursing profession nationally and internationally, which is further compounded by the universal issues of inadequate working conditions, high turnover rates and global nurse shortages. High workplace demands such as role strain and work overload may lead to negative outcomes for nurses, including burnout and intent to leave the profession (Jourdain & Chênevert, 2010; Lavoie‐Tremblay, Trepanier, Ferner, & Bonneville‐Roussy, 2013; Spence Laschinger, Grau, Finegan, & Wilk, 2012). Studies have examined autonomy, job control and collegial/managerial support and found that these types of resources have an impact on motivational processes leading to a higher degree of work engagement and organizational commitment among nurses (Mauno, Kinnunen, & Ruokolainen, 2007; Nahrgang, Morgeson, & Hofmann, 2011). There appears to be a relationship between various job demands/resources and nurses’ overall well‐being in their workplaces. With few exceptions (Lenthall et al., 2018), the majority of these studies involving nurses have been hospital‐based and use a limited set of researcher‐defined indicators of both job demands and resources, with less reliance on psychometrically sound and integrated measures. This article describes the development, pilot testing and psychometric evaluation of two new scales measuring the global (i.e., applicable to a broad range of nursing designations and practice settings) Job Resources in Nursing (JRIN) and Job Demands in Nursing (JDIN) as part of a nationwide cross‐sectional survey involving nurses in a wide range of workplaces in rural and remote communities.

## 2 BACKGROUND

The Job Demands–Resources (JD‐R) Model integrates both the occupational stress and motivational research traditions (Bakker, Demerouti, & Schaufeli, 2003; Demerouti & Bakker, 2011; Demerouti, Bakker, Nachreiner, & Schaufeli, 2001). This model assumes that occupational groups have particular features that are associated with job‐related stress, classified into two main categories: job demands and job resources (Bakker, Demerouti, & Euwema, 2005; Bakker, Demerouti, De Boer, & Schaufeli, 2003; Demerouti & Bakker, 2011). Job demands are defined as the physical, psychological, social and organizational aspects of employment that contribute to sustained cognitive and emotional effort, leading to job‐related stress. Job resources are viewed as the physical, psychological, social and organizational aspects of employment that are thought to foster motivation to achieve work‐related goals and personal growth and buffer the impact of job‐related demands (Bakker & Demerouti, 2007; Bakker, Hakanen, Demerouti, & Xanthopoulou, 2007; Llorens, Bakker, Schaufeli, & Salanova, 2006).

Various combinations of job demands and job resources in relation to occupational outcomes in nursing and other service professions have been explored. These include organizational commitment and intent to leave (Jourdain & Chênevert, 2010; Rudman, Gustavsson, & Hultell, 2014; Schmidt & Diestel, 2013), work engagement (Bakker et al., 2007; Schaufeli & Bakker, 2004), burnout (Alarcon, 2011; Bakker et al., 2005; Demerouti, Bakker, Nachreiner, & Schaufeli, 2000) and professional well‐being (Boudrias et al., 2011; Spence Laschinger et al., 2012). Research suggests that increased job demands for nurses have adverse effects of higher emotional exhaustion, greater psychosomatic complaints and intent to leave (Schmidt & Diestel, 2013). Common job demands in nursing that have shown similar directional relationships with these outcomes include high workloads, lack of safe working conditions and unfavourable work environments (Al‐Homayan, Shamsudin, Subramaniam, & Islam, 2013; Jourdain, & Chênevert, 2010; Schmidt & Diestel, 2013).

Research involving nurses also suggests that having a sufficient amount of varied job resources can predict lower distress and safeguard against high job demands in the workplace (Lavoie‐Tremblay et al., 2013). Job resources in nursing that are shown to potentially have an impact on outcomes are broad‐ranging and include those at the organizational level (e.g., staffing, career opportunity and professional development; Carter & Tourangeau, 2012), the interpersonal (e.g., collegial support; Van den Tooren & Jonge, 2008) and position/task level (e.g., autonomy and performance feedback; Islam & Al‐Homayan, 2013; Mauno et al., 2007).

Most studies exploring job demands and job resources in the context of nursing practice have used various combinations of single items, portions of standardized scales and/or instruments focused primarily on the acute care, urban and organizational level such as the Nursing Work Index‐Revised (Aiken & Patrician, 2000). To improve important occupational outcomes such as retention and work engagement and reduce levels of burnout, it is necessary to develop a multidimensional conceptualization of the most salient job demands and job resources across areas of nursing practice. These instruments should have the ability to conceptually measure various job demands and job resources applicable to nursing practice as a whole, while allowing for potential comparisons to be made across nursing designations (e.g., registered nurses [RNs], nurse practitioners [NPs], licensed practical nurses [LPNs], registered psychiatric nurses [RPNs]), practice settings (e.g., acute care and public health) and geographical locations (e.g., rural and urban).

## 3 THE STUDY

### 3.1 Aim

The overall aim of this project was to develop and test the psychometric properties of the Job Resources in Nursing (JRIN) Scale and the Job Demands in Nursing (JDIN) Scale. To achieve this aim, the following objectives were employed: (a) development of two new scales measuring the Job Resources and the Job Demands of nurses who practice in a variety of roles and content evaluation with item‐by‐item verification; (b) assessing the psychometric properties and reducing the length of the JRIN and JDIN scales through a pilot survey of Canadian nurses with experience in rural and remote area practice; and (c) further test the factor structure of each scale through exploratory factor analysis of data from a Pan‐Canadian survey of RNs, NPs, LPNs and RPNs.

### 3.2 Design

In accordance with our aim and stated objectives, we employed a cross‐sectional design with a three‐phase process of instrument development and evaluation. Phase 1 involved an extensive literature review, expert consultation and an iterative content evaluation to develop the initial scale items. Phase 2 involved a pilot survey of 89 nurses and use of item discrimination analysis to estimate the internal consistency reliability of each subscale and reduce the length of each scale (data collected between September and December 2013). Phase 3 consisted of a nationwide cross‐sectional survey of 3,822 rural and remote nurses to further test the modified scales and use of exploratory factor analysis to finalize the factor structure of both scales (data collected between April 2014 and September 2015).

### 3.3 Sample/participants

Those who were eligible to participate in the pilot survey included RNs, NPs, LPNs and RPNs practicing in Canada with recent/current experience in rural or remote settings. Based on power analyses (Bonett, 2002), the target sample size for internal consistency reliability testing of a 42‐ to 60‐item scale (desired Cronbach's alpha [*α*] of 0.80) was 100 participants. Snowball sampling was used to recruit Phase 2 study participants (*N* = 89) through emails with study information and a direct link to the online survey. For the Phase 3 national cross‐sectional survey, a stratified, systematic sample of 10,072 RNs, LPNs, RPNs and NPs was initially targeted, with 9,622 eligible participants in rural and remote communities across all Canadian provinces and territories. There were 450 participants who were ineligible due to incorrect addresses, duplicate registrations or were retired. A total of 3,822 participants completed surveys via mailed paper version or secure online access, for a response rate of 40%.

A total of 2,774 participants completed all 24 items related to job resources, and 2,431 participants completed all 24 items related to job demands. Only participants with valid responses on each item in both scales were included in the exploratory factor analysis to reduce artificially high correlations resulting from imputation of missing values (Tabachnick & Fidell, 2013). For the job resources analysis, there were no significant differences between those included and excluded (*N* = 1,081) in the analysis based on gender or nursing registration status (i.e., RN, NP, LPN and RPN), with included participants being slightly younger (mean: 46.6, *SD*: 11.7) than those excluded (mean: 48.9, *SD*: 11.9, *p* < 0.0001). For the job demands analysis, there were no differences based on nursing registration status and slight differences in gender (93.1% women in the included vs. 94.8% in the excluded group [*N* = 1,481]), with included participants being slightly younger (mean: 46.9, *SD*: 11.8) than those excluded (mean: 48.2, *SD*: 11.5, *p* < 0.001).

## 4 INSTRUMENT

### 4.1 Construct validity testing

Although three distinct types of validity (i.e., content, criterion and construct) have traditionally been identified, Cook and Beckman (2006) questioned these distinctions and proposed the conceptualization of “construct validity” as an overarching framework. Five evidence sources that support construct validity are content, response process, internal structure, relation to other variables and consequences (Messick, 1989 as cited in Cook & Beckman, 2006). Four sources of construct validity evidence were drawn on through the psychometric testing process. Phase 1 instrument development and Phase 2 pilot testing provided validity evidence of both content (e.g., evaluation of the process for developing and selecting items) and response process (e.g., methods for scoring and reporting results). Internal structure and relation to other variables were further tested in Phase 3 using exploratory factor analysis and examination of the correlation of scale scores with the scores from other concepts measured (e.g., work engagement and burnout) in the Phase 3 national survey.

### 4.2 Reliability testing

Internal consistency reliability was examined using Cronbach's alpha estimates during Phase 2 pilot testing to assess how well individual item related to other items and “Cronbach's alpha if deleted” to assess the item contribution to each subscale and full scales. Cronbach's alpha estimates were repeated for the Phase 3 national survey data to confirm the internal consistency reliability for the final versions of both scales.

### 4.3 Ethical considerations

Research Ethics Committee approval was attained from the separate research ethics boards of each of the research team members’ institutions prior to the Phase 2 pilot survey testing and Phase 3 national survey.

### 4.4 Phase 1: Instrument development

The first phase included an extensive literature review and discussions with a national 16‐member research team (including nine registered nurses, four nurse practitioners, a geographer, a statistician and a psychiatric epidemiologist) to determine key dimensions related to job resources and job demands in nursing. Essential research that guided this process was a national survey of the nature of nursing practice in rural and remote Canada (Stewart et al., 2005) and the work completed in Australia exploring resources and demands in rural and remote nursing practice (Lenthall et al., 2011, 2009). In total, seven key dimensions of job resources in nursing (JRIN) and 10 key dimensions of job demands in nursing (JDIN) were identified by the research team. The job resources dimensions were as follows: (a) supervision, recognition and feedback; (b) collegial support; (c) staffing and time; (d) technology; (e) training; professional development and continuing education; (f) autonomy and control; and (g) support from care recipient and their family members. The job demands dimensions were as follows: (a) work‐related travel; (b) on‐call; (c) rural healthcare ethics; (d) preparedness/extended scope of practice; (e) cultural issues; (f) equipment and supplies; (g) isolation; (h) workload; (i) scheduling; and (j) safety.

Next, the scale developers (K.L.P & J.G.K) generated six items for each of the seven job resources (42 items total) and 10 job demands (60 items total) subscales, with each subscale representing a key dimension. Item development was informed by studies that addressed the key dimensions identified (Crowden, 2010; Delobelle et al., 2011; DesRoches, Miralles, Buerhaus, Hess, & Donelan, 2011; Hanvey, 2005; Hayes et al., 2006; Hunsberger, Baumann, Blythe, & Crea, 2009; Lenthall et al., 2011, 2009; Nelson, Pomerantz, Howard, & Bushy, 2007; Orchard, King, Khalili, & Bezzina, 2012; Penz et al., 2007; Penz, Stewart, & D'Arcy, & Morgan, D., 2008; Stewart et al., 2005; Thompson, 2004). A draft version of both scales was then provided to our 19‐member national advisory team (i.e., nursing leaders and policymakers in each province and territory) who worked with the research team through an iterative process (i.e., items revised and added/excluded) to further verify the content validity of both scales. This was achieved through a combination of a two full day sessions and seven teleconferences, with the research and advisory teams providing conceptual and item‐specific feedback, until consensus was reached on the content, phrasing and format of three positively worded (reverse scored for the JDIN) and three negatively worded items (reverse scored for the JRIN) distributed randomly in each subscale. Both scales were scored on a five‐point Likert scale from: 1 (strongly disagree); 2 (disagree); 3 (neutral); 4 (agree); to 5 (strongly agree); or 97 (not applicable). “Not applicable” responses were coded as “missing.” Higher JRIN scores indicated a higher level of job resources, and higher JDIN scores indicated a higher level of job demands.

### 4.5 Phase 2: Pilot survey testing

A pilot survey involving 89 nurses was conducted, which included evaluation of the initial psychometric properties of and refine/reduce the items included in both scales. The pilot survey questionnaire (15 pages in the paper version) consisted of questions about demographics, employment and work–community/setting, with the key purpose of testing the psychometric properties of four scales: the 42‐item Job Resources in Nursing (JRIN) Scale, the 60‐item Job Demands in Nursing (JDIN) Scale, an adapted 12‐item Work Satisfaction Scale (Williams et al., 1999) and a newly developed 60‐item Primary Health Care Engagement Scale (the results of which are published elsewhere, see Kosteniuk et al., 2016, 2017). Demographic characteristics of the pilot survey sample were analysed using IBM SPSS, v23.0. Cronbach's alpha coefficients were used to evaluate the internal consistency reliability of each subscale and the overall scales. Prior to performing reliability estimates for each subscale, case means were imputed for missing values (El‐Masri & Fox‐Wasylyshyn, 2005). When a participant's subscale was missing 25% or less items (i.e., one item), case mean imputation for missing values was conducted for each subscale. If more than 25% of the items were missing, the subscale score was discarded (El‐Masri & Fox‐Wasylyshyn, 2005). The item discrimination method (Furr & Bacharach, 2008) and the conceptual judgement of our research data team were used to evaluate the corrected item‐total correlation (*r*) for each item. The range in item‐total correlation was evaluated as lower than 0.30 (weak), 0.30–0.49 (moderate) and ≥0.50 (substantial; Kellar & Kelvin, 2013). The items with the lowest item‐total correlation were removed, with reliability estimates calculated after each removal. The aim was to identify the four items that had the strongest association to represent the construct. The internal consistency reliability was estimated for shortened version of each subscale. Cronbach's alpha of 0.70 indicated modest or acceptable internal consistency reliability for each subscale (Nunnally & Bernstein, 1994). Through the item analysis process described in the pilot testing results, decisions were also made to remove one JRIN subscale and four JDIN subscales, leaving a 24‐item JRIN Scale and a 24‐item JDIN Scale for further evaluation.

### 4.6 Phase 3: National cross‐sectional survey: exploratory factor analysis

The data used to analyse the factor structure of the JRIN and JDIN scales were from a nationwide cross‐sectional study, the Nature of Nursing Practice in Rural and Remote Canada II involving 3,822 registered nurses (RNs), nurse practitioners (NPs), licensed or registered practical nurses (LPNs) and registered psychiatric nurses (RPNs; MacLeod et al., 2017). The 27‐page national survey questionnaire included five main sections of individual, community, workplace, nursing practice and personal/professional well‐being and was designed to develop a better understanding of factors influencing the occupational outcomes for nurses in rural/remote communities across Canada (MacLeod et al., 2017). Descriptive statistics were used to analyse the demographic characteristics of the national level data for the JRIN and the JDIN factor analysis subsamples. Factor analysis is a “large‐sample” procedure where generalizability of results is unlikely if the study sample is too small (Osborne & Costello, 2009), which was the case in the pilot testing phase of this study. The sample size of 2,774 (JRIN analysis) and 2,341 (JDIN analysis) was more than adequate (Comrey & Lee, 1992). IBM SPSS v23.0 was used to conduct exploratory factor analysis of the 24 items related to job resources and the 24 items related to job demands. Principal component method of extraction and varimax rotation (Tabachnik & Fidell, 2013) were used to analyse the factor structure of both scales. Factorability was evaluated with Kaiser–Meyer–Olken (KMO) measure of sampling adequacy (considered acceptable if >0.75–0.80) and Bartlett's Test of Sphericity (significance values <0.05 indicating suitability for factor structure detection). The quality of the final solution for both the JRIN and JDIN was confirmed by the amount of variance explained, item loadings ≥0.40, Scree plot evaluation, eigenvalues >1.0 and the conceptual fit of the resulting factors. The item scores for each of the dimensions in both the JRIN and JDIN were summated then divided by the total number of items in each subscale to produce a mean score on each. Scores of 1.0–3.0 indicate a low degree of agreement and scores of >3.0 are suggestive of perceived high agreement on that dimension or subscale. Internal consistency reliability using Cronbach's alpha coefficients was calculated for each subscale and the full scales. Finally, to further analyse construct validity (i.e., relation to other variables, Messick, 1989 as cited in Cook & Beckman, 2006), correlation of scale scores with the scores from other concepts measured (e.g., work engagement and burnout) was also calculated using the national survey data.

## 5 RESULTS

### 5.1 Pilot sample characteristics

Pilot testing involved 89 survey respondents of which 92.1% were female (*N* = 82). The average age of respondents was 44.8 (*SD*: 12.4), with a range in age from 24‐82 (study included those who were retired but occasionally employed in nursing). The majority of respondents worked in British Columbia (58.4%), Saskatchewan (16.9%) and Alberta (9%), with the remaining 15.7% working in Ontario, Nova Scotia, New Brunswick, Newfoundland and Labrador, the Yukon and the Northwest Territories. Respondents were RNs (88.8%), NPs (15.7%), RPNs (2.2%) and LPNs (3.4%) and were employed in a variety of settings (e.g., hospital, home care and community health). Most respondents were direct care providers (67.4%), held positions of leadership (11.2%) or worked in nursing education/other (17.9%).

### 5.2 Pilot testing: item analysis and scale reduction

Table 1 outlines the item‐total correlations (range in values from −0.07 to 0.86) and reliability estimates for the original seven 6‐item JRIN subscales. For the JRIN items, a total of two items correlated weakly (<0.30), 12 items correlated moderately (0.30–0.49) and the remaining 28 correlated strongly (≥0.50). To shorten the length of the overall JRIN scale and ensure applicability of subscale items across nursing designations, a team decision was made to remove “Support from Care Recipients and their Family Members” subscale. In the remaining six JRIN subscales, the items with the lowest item‐total correlations were removed one at a time, with reliability estimates repeated after each removal to achieve the highest alpha with the smallest number of items.

**Table 1:** Pilot testing: Job Resources in Nursing (JRIN) dimensions (subscales) and item analysis (*N* = 89)

Item	*M*	*SD*	*r*	Cronbach's *α* if item deleted
A. Supervision, recognition and feedback				
**1. I receive adequate praise and recognition from my supervisor for the work that I do**	3.0	1.3	0.83	0.82
2. I do not have a clear understanding about what is expected of me in my nursing role[Link]	3.6	1.0	0.41	0.89
**3. My supervisor is concerned with my welfare and the welfare of my colleagues working in this setting**	3.2	1.1	0.65	0.85
4. I feel like my supervisor does not trust my judgement regarding my practice[Link]	2.9	0.9	0.61	0.81
**5. The feedback from my supervisor about the work that I do is not adequate** [Link]	3.2	1.2	0.72	0.84
**6. I feel validated by my supervisor for a job well done**	3.1	1.2	0.86	0.81
B. Collegial support				
1. I do not feel like my colleagues and support staff are helpful to me when I need assistance[Link]	3.9	1.0	0.54	0.84
**2. There is a sense of mutual trust and respect between my colleagues and myself**	3.8	1.0	0.75	0.80
**3. I do not feel like I can rely on my colleagues to help me when I am having a difficult time at work** [Link]	3.9	1.0	0.76	0.79
4. I would consider the communication between staff members in this setting to be open and honest	3.6	1.0	0.73	0.80
**5. I do not feel like my colleagues are competent in their roles** [Link]	3.9	1.0	0.33	0.87
**6. I feel supported by my colleagues for the work that I do as a nurse**	3.9	0.9	0.73	0.80
C. Staffing and time				
**1. There are enough staff members in my work setting to get the job done**	2.7	1.2	0.63	0.73
**2. The nursing staff mix in my work setting is appropriate**	3.2	1.0	0.62	0.74
3. My workplace is not able to accommodate the vacation time that I request[Link]	3.5	1.0	0.30	0.81
**4. There are not enough support staff in my work setting** [Link]	2.7	1.2	0.66	0.73
5. The time that I spend on non‐nursing duties is reasonable	2.7	1.1	0.45	0.78
**6. I do not have enough time to do what is important (beyond just basic care) in my nursing role** [Link]	3.1	1.1	0.62	0.74
D. Technology				
1. I do not have a good understanding of how to operate the medical technology required to do my job[Link]	4.1	0.8	−0.07	0.67
2. I have adequate internet access for use in my nursing practice	3.9	0.9	0.06	0.63
**3. I am not satisfied with the availability of electronic communication with my co‐workers** [Link]	3.5	1.1	0.39	0.51
**4. I do not feel that my access to electronic patient information is adequate** [Link]	3.0	1.3	0.48	0.46
**5. My access to electronic resources (e.g., ordering of tests, access to patient information and results) is adequate for my nursing practice**	3.0	1.2	0.57	0.42
**6. I am able to provide better care because of the information systems and technology available to me**	3.0	1.1	0.53	0.45
E. Training, professional development and continuing education				
**1. I am able to access an adequate number of in‐services or continuing education activities**	3.3	1.1	0.54	0.75
**2. I do not receive adequate training on new technology** [Link]	3.4	1.0	0.54	0.75
3. The orientation provided by my primary work setting adequately prepared me for my current nursing role	2.9	1.2	0.41	0.78
**4. I do not have enough opportunities for professional growth and development** [Link]	3.3	1.2	0.67	0.71
**5. I do not feel that a sufficient amount of training is provided when I take on new nursing responsibilities and/or competencies** [Link]	3.1	1.0	0.68	0.71
6. The information that I learn during in‐services or continuing education activities is helpful in my nursing role	3.9	0.6	0.44	0.77
F. Autonomy and control				
**1. I do not feel like I have an adequate amount of decision‐making freedom in my nursing role** [Link]	3.7	1.1	0.62	0.83
**2. My job description is flexible (I am able to modify my daily duties or the type of work that I do)**	3.6	1.0	0.67	0.82
**3. I do not feel that I have direct influence on shaping my work environment and how care is provided in my work setting** [Link]	3.4	1.1	0.73	0.81
**4. Often I feel like I am not allowed to use my professional nursing judgement to act in my patient's best interest** [Link]	3.5	1.1	0.77	0.80
5. I have the independence to make decisions that exceed usual nursing practice	3.5	1.0	0.41	0.87
6. I am proud that I am able to work autonomously in my role as a nurse in this setting	3.9	0.9	0.70	0.82
G. Support from care recipients and their family members				
1. I feel motivated by the recognition that I receive from patients and their families	4.1	0.7	0.34	0.71
2. I am not satisfied with the level of closeness between myself, patients and their family members[Link]	3.6	1.0	0.42	0.70
3. I do not feel like patients and their family members appreciate my role as a nurse[Link]	3.9	0.9	0.50	0.67
4. My interpersonal interactions with patients and their family members seem to offset some of my work‐related challenges	3.5	0.9	0.39	0.70
5. The level of support and assistance I receive from the family members of patients is not adequate[Link]	3.4	0.9	0.59	0.65
6. I am comfortable with knowing patients and their family members personally and in a sense I feel like I am caring	3.7	1.0	0.53	0.66

Note

Bolded items were retained in the 24‐item JRIN Scale for further testing.

*M*: mean; *r*: corrected item‐total correlation; *SD*: standard deviation.

Reverse scored.

Table 2 outlines the item‐total correlations and reliability estimates for the original JDIN scale consisting of ten 6‐item subscales. The item‐total correlations ranged from −0.19‐0.80, with 14 items correlating weakly (<0.30), 20 items moderately (0.30–0.49) and the remaining 26 correlating strongly (≥0.50). Three subscales with lower alphas (on‐call [*α* = 0.69], rural healthcare ethics [*α* = 0.54] and cultural issues [*α* = 0.61]) were removed from the JDIN scale. Although the “Scheduling” subscale returned strong item‐total correlations, a team decision was made to remove this subscale due to theoretical overlap with national survey items and to broaden the applicability (e.g., not all nurses work shift work). The steps of removing the items with the lowest item‐total correlation one at a time and examining reliability estimates after each removal were performed for the remaining six JDIN subscales.

**Table 2:** Pilot testing: Job Demands in Nursing (JDIN) dimensions (subscales) and item analysis (*N* = 89)

Item	*M*	*SD*	*r*	Cronbach's *α* if item deleted
A. Work‐related travel				
**1. Travelling for my work does not interfere with my family life** [Link]	2.8	1.3	0.68	0.77
**2. Travelling for my work is tiring**	3.0	1.2	0.68	0.77
**3. I feel that my health has suffered because of my work‐related travel**	2.3	1.0	0.70	0.77
**4. I am satisfied with the amount of travel involved in my work** [Link]	2.7	1.1	0.68	0.77
5. I generally feel safe even when I have to travel in difficult weather conditions[Link]	2.87	1.1	0.34	0.84
6. If I had a choice I would travel less as part of my work	3.00	1.1	0.49	0.81
B. On‐call				
1. I am satisfied with the amount of time that I am on‐call[Link]	2.5	1.0	0.68	0.56
2. If I had a choice I would be on‐call less often	3.3	1.0	0.59	0.59
3. I am seldom called out for non‐urgent issues[Link]	2.8	1.3	0.29	0.69
4. I am asked to be on‐call on my days off	3.2	1.2	0.08	0.75
5. Even when I'm not officially on‐call, I still feel like I am on duty	2.6	1.2	0.62	0.56
6. I am not asked to be on‐call when I am unwell[Link]	2.5	1.1	0.34	0.66
C. Rural healthcare ethics				
1. It is an ongoing challenge to protect patient confidentiality (e.g., from staff or community members)	3.5	1.1	0.32	0.42
2. I feel like I am able to say “no” when people ask me for my advice when I'm not at work[Link]	3.0	1.0	0.26	0.46
3. I feel that my privacy is respected in the community[Link]	2.6	1.0	0.26	0.41
4. I feel that patient care is compromised by limited access to some healthcare services	3.7	1.0	0.11	0.53
5. It is easy to separate my role as a nurse and my other roles in the community[Link]	2.9	1.0	0.29	0.44
6. When I'm working, patients treat me too much like a friend and not enough like a professional	2.1	0.7	0.25	0.47
D. Preparedness/extended scope of practice				
**1. I am satisfied with my day‐to‐day routine** [Link]	2.1	0.6	0.60	0.57
**2. I do not feel adequately prepared for my area of practice**	2.0	0.8	0.60	0.55
**3. I feel that I have the necessary knowledge to do my work** [Link]	1.9	0.6	0.73	0.52
**4. I feel that I have the necessary skills to do my work** [Link]	1.9	0.6	0.71	0.54
5. I feel responsible for my own continuing education	4.1	0.7	−0.19	0.80
6. I feel pressured to work beyond my scope of practice	2.2	0.9	0.28	0.69
E. Cultural issues				
1. I have experienced prejudice, discrimination or racism in my current position	2.8	1.3	0.27	0.63
2. I find it difficult to work with patients in the community due to cultural differences	1.9	0.7	0.41	0.48
3. I have a good understanding of the values and beliefs of the community[Link]	1.9	0.6	0.32	0.56
4. I received adequate cultural sensitivity training (e.g., during educational preparation, at work)[Link]	2.6	1.1	0.13	0.58
5. The values and beliefs of the community make it more difficult to do my work	2.2	1.1	0.41	0.48
6. I can appreciate my patients’ cultural values and beliefs, although they may be different than my own values and beliefs[Link]	1.6	0.5	0.16	0.61
F. Equipment and supplies				
**1. The supplies that I require for my work are at‐hand when I need them** [Link]	2.4	1.0	0.61	0.46
**2. The equipment that I need to do my work is readily available** [Link]	2.5	1.1	0.60	0.47
3. I am responsible for maintaining non‐medical workplace equipment (e.g., vehicle and generator)	2.6	1.1	0.11	0.68
**4. The equipment needed for patient care is poorly maintained**	2.3	0.9	0.43	0.55
**5. I know how to use the necessary equipment for patient care** [Link]	1.8	0.5	0.17	0.63
6. I am responsible for ordering supplies	3.3	1.2	0.30	0.61
G. Isolation				
1. I would prefer working closer to services (e.g., businesses, government services and recreation facilities)	3.0	1.0	0.37	0.71
**2. I have opportunities to debrief with others after difficult experiences** [Link]	2.4	1.0	0.47	0.68
3. I feel a sense of isolation from family and friends	2.7	1.3	0.44	0.69
**4. I am comfortable working alone** [Link]	2.2	0.9	0.49	0.67
**5. I feel a sense of isolation from my colleagues**	2.4	1.0	0.60	0.64
**6. I have colleagues I can call on for back‐up** [Link]	1.9	0.7	0.42	0.70
H. Workload				
**1. There is not enough time in my day to do what I am expected to do**	3.2	1.1	0.52	0.72
**2. I am comfortable with the amount of mental effort required for my work** [Link]	2.4	0.9	0.60	0.70
**3. I am comfortable with the amount of physical effort required for my work** [Link]	2.2	0.8	0.43	0.74
4. I can choose the pace at which I work[Link]	3.0	1.1	0.49	0.72
**5. I feel like I work more hours than I am paid for**	3.1	1.2	0.61	0.69
6. My work interferes with family obligations	2.8	1.0	0.40	0.75
I. Scheduling				
1. The number of hours I work each month is satisfactory[Link]	2.1	0.8	0.50	0.85
2. I am able to change my assigned shifts when I need to[Link]	2.8	1.1	0.66	0.82
3. I am not satisfied with the shifts I work	2.3	0.9	0.73	0.81
4. I would like to have more flexibility in overall scheduling[Link]	3.1	1.2	0.59	0.84
5. I am satisfied with my shift rotation	2.4	1.0	0.80	0.79
6. I am not satisfied with the amount of overtime I work	2.5	0.8	0.62	0.83
J. Safety				
**1. There are times that I feel that my personal safety is at risk in my workplace**	2.8	1.2	0.47	0.69
2. Other healthcare providers in my workplace face a low level of safety risk in their everyday work[Link]	2.9	1.0	0.34	0.72
**3. There are times that I feel that my personal safety is at risk when I am off‐duty**	2.2	1.0	0.51	0.68
**4. Working in a rural/remote setting does not pose a personal safety risk for me** [Link]	2.5	1.1	0.60	0.65
5. At times I am concerned for the safety of my patients or their family members	3.4	0.9	0.30	0.73
**6. My workplace responds appropriately to staff's safety concerns** [Link]	2.4	1.0	0.59	0.65

Note

Bolded items were retained in the 24‐item JDIN Scale for further testing.

*M*: mean; *r*: corrected item‐total correlation; *SD*: standard deviation

Reverse scored.

Reflected in Table 3 are the refined JRIN Scale dimensions following pilot testing namely supervision, collegial support, staffing, technology, professional development and autonomy and control. Table 4 shows the refined JDIN Scale dimensions, which include work‐related travel, preparedness/extended scope of practice, equipment and supplies, isolation, workload and safety. The mean, standard deviation, range and internal consistency reliability are indicated for the initial 6‐item and final 4‐item JRIN and JDIN subscales. The newly refined 4‐item subscales for both the JRIN and JDIN scales were embedded into the national survey questionnaire for further analysis.

**Table 3:** Mean scores and internal consistency reliability of the 42‐item Job Resources in Nursing (JRIN) Scale 6‐item and 4‐item refined subscales based on pilot testing

Subscale	6‐item subscales				4‐item subscales			
	*N*	Mean score (*SD*)	Range	Cronbach's *α*	*N*	Mean score (*SD*)	Range	Cronbach's *α*
Supervision, recognition and feedback	80	20.2 (5.5)	6–30	0.87	80	12.7 (4.4)	4–20	0.90
Collegial support	81	23.1 (4.6)	11–30	0.85	81	15.3 (3.5)	5–20	0.89
Staffing and time	78	18.1 (4.8)	8–29	0.79	78	11.8 (3.8)	4–20	0.82
Technology	72	20.8 (3.9)	12–30	0.59	72	12.8 (3.6)	5–20	0.73
Training, professional development and continuing education	78	20.2 (4.5)	9–30	0.79	79	13.2 (3.6)	5–20	0.79
Autonomy and control	78	21.8 (4.9)	6–30	0.86	79	14.2 (3.9)	3–20	0.87
Job Resources in Nursing Scale (all six subscales)	69	123.5 (20.4)	73–172	0.90	69	79.5 (15.8)	42–114	0.91

Note

*SD*: standard deviation.

**Table 4:** Mean scores and internal consistency reliability of the 60‐item Job Demands in Nursing (JDIN) Scale 6‐item and 4‐item refined subscales based on pilot testing

Dimension/Subscale	6‐item subscales				4‐item subscales			
	*N*	Mean score (*SD*)	Range	Cronbach's *α*	*N*	Mean score (*SD*)	Range	Cronbach's *α*
Work‐related travel	71	16.8 (5.1)	6–30	0.82	71	10.9 (3.9)	4–20	0.85
Preparedness/extended scope of practice	77	14.6 (2.7)	7–23	0.71	78	8.1 (2.3)	4–16	0.87
Equipment and supplies	67	15.1 (3.7)	6–23	0.63	71	9.2 (2.9)	4–16	0.75
Isolation	74	14.8 (4.0)	8–30	0.73	74	9.1 (2.8)	4–20	0.72
Workload	79	16.9 (4.4)	9–27	0.76	79	11.0 (3.1)	4–19	0.73
Safety	76	15.6 (4.2)	7–28	0.73	78	10.1 (3.3)	4–20	0.74
Job Demands in Nursing (JDIN) Scale (all subscales)	57	94.4 (15.8)	57–129	0.89	62	57.9 (11.5)	33–85	0.87

Note

*SD*: standard deviation.

### 5.3 National survey data: factor analysis sample characteristics

Table 5 outlines the demographic characteristics of participants included in the factor analysis of the national level data: all valid responses for the JRIN (*N* = 2,774) and the JDIN (*N* = 2,341) factor analyses. The majority of participants were female and ranged in age from 19 to 84 years (study included those who were retired but occasionally employed in nursing), with an average age of 46.8 years. Just over half of each sample was RNs, with the one in three LPN participants. Participants were distributed across Canada, with the majority indicating “staff nurse” as their primary position. A diversity of practice settings was represented, with the majority of nurses working in acute care, long‐term care, community health and primary care.

**Table 5:** National survey demographic characteristics JRIN and JDIN factor analyses

Characteristic	JRIN sample (*N* = 2,774) *N* (%)	JDIN sample (*N* = 2,341) *N* (%)
Nurse type		
Registered nurse (RN)	1,527 (55.0)	1,273 (54.4)
Nurse practitioner (NP)	124 (4.5)	115 (4.9)
LPN/Registered practical nurse	972 (35.0)	831 (35.5)
Registered psychiatric nurse	151 (5.4)	122 (5.2)
Gender[Link]		
Female	2,501 (90.2)	2,097 (89.6)
Male	169 (6.1)	156 (6.7)
Age[Link]		
<30 years old	240 (8.7)	213 (9.1)
30–39 years old	519 (18.7)	429 (18.3)
40–49 years old	624 (22.5)	514 (22.0)
50–59 years old	903 (32.6)	746 (32.0)
60+ years old	356 (12.8)	321 (13.7)
Province of primary employment		
Atlantic provinces (i.e., New Brunswick, Nova Scotia, Newfoundland and Labrador)	702 (25.3)	581 (24.8)
Manitoba/Saskatchewan	597 (21.5)	525 (22.4)
Alberta/British Columbia	506 (18.2)	423 (18.0)
Yukon and North West Territories	453 (16.3)	356 (15.2)
Ontario	317 (11.4)	244 (10.4)
Quebec	199 (7.2)	212 (9.1)
Primary position[Link]		
Staff nurse	2,231 (80.4)	1,835 (78.4)
NP/CNS	215 (7.6)	192 (8.2)
Manager	203 (7.3)	184 (7.9)
Educator/Consultant	62 (2.2)	68 (2.9)
Employment status[Link]		
Full‐time	1,491 (53.7)	1,203 (51.4)
Part‐time/Job share/Casual/Contract/Term)	1,417 (51.1)	1,252 (53.4)
Area of current practice[Link]		
Acute care	1,293 (46.6)	1,002 (42.8)
Long‐term care	813 (29.3)	697 (29.8)
Community health	387 (13.9)	377 (16.1)
Primary care	348 (12.5)	316 (13.5)
Mental health	278 (10.0)	222 (9.5)
Home care	257 (9.3)	276 (11.8)
Palliative/End‐of‐life care	207 (7.5)	202 (8.6)
Have dependent children or adults in their care [Link]		
Yes	1,306 (47.1)	1,096 (47.8)
No	1,453 (52.4)	1,229 (52.5)

May not add up to 100% based on missing data.

May hold more than one employment status designation.

May work in more than one area of practice.

### 5.4 Exploratory factor analysis and mean subscale scoring

The JRIN and JDIN scales were examined further for construct validity with exploratory factor analysis of data from the full national survey. Table 6 outlines the final subscale dimensions, factor loadings, overall mean subscale scores and reliability for the JRIN subscales and full scale. Factorability of the 24 items related to job resources in the national data was confirmed through a significant Bartlett's Test of Sphericity (*χ* ^2^ = 27,049.368, *df* = 276, *p* < 0.0001) and an acceptable Kaiser‐Meyer‐Olkin measure of sampling adequacy (KMO) of 0.878. Exploratory factor analysis was then performed on the 24 items related to job resources in nursing, which favoured a 6‐factor structure (four items per factor) identical to the original subscale conceptualizations developed/tested during Phase 1 and Phase 2. All factor loadings were >0.40, ranging from 0.55 to 0.87 (no cross‐loadings) for all 24 items, with eigenvalues >1.0 (range from 1.24‐6.68), together explaining 63% of the variance. The subscale dimensions based on factor loadings for the JRIN scale were: (a) supervision, recognition and feedback; (b) training, professional development and continuing education; (c) collegial support; (d) staffing and time; (e) technology; and (f) autonomy and control, with scores ranging from 1 (strongly disagree) ‐ 5 (strongly agree). The mean score on each dimension of the JRIN ranged from 2.9‐4.0, with the lowest perceived job resources related to Staffing and Time (mean: 2.9, *SD*: 0.9) to the highest job resources related to Collegial Support (mean: 4.0, *SD*: 0.7). Good internal consistency reliability was noted for the JRIN subscales (*α* = 0.74–0.88) and the full 24‐item JRIN scale (*α* = 0.88) for the national sample analysis.

**Table 6:** Factor structure, reliability and mean scores of the final 24‐item Job Resources in Nursing (JRIN) Scale (*N* = 2,774)

Job resources factors	Mean (*SD*)	Factor loadings	Cronbach's *α*
Supervision, recognition and feedback			
1. I receive adequate praise and recognition from my supervisor for the work that I do	3.2 (1.0)	0.87	0.88
2. I feel validated by my supervisor for a job well done		0.87	
3. My supervisor is concerned with my welfare and the welfare of my colleagues working in this setting		0.79	
4. The feedback from my supervisor about the work that I do is not adequate[Link]		0.70	
Training, professional development and continuing education			
1. I do not have enough opportunities for professional growth and development[Link]	3.2 (0.9)	0.82	0.84
2. I am able to access an adequate number of in‐services or continuing education activities		0.78	
3. I do not receive adequate training on new technology[Link]		0.77	
4. I do not feel that a sufficient amount of training is provided when I take on new nursing responsibilities and/or competencies[Link]		0.72	
Collegial support			
1. There is a sense of mutual trust and respect between my colleagues and myself	4.0 (0.7)	0.79	0.77
2. I feel supported by my colleagues for the work that I do as a nurse		0.78	
3. I do not feel that I can rely on my colleagues to help me when I am having a difficult time at work[Link]		0.75	
4. I do not feel like my colleagues are competent in their roles[Link]		0.68	
Staffing and time			
1. There are enough staff members in my work setting to get the job done	2.9 (0.9)	0.83	0.78
2. I do not have adequate time to do what is important (beyond just basic care) in my nursing role[Link]		0.74	
3. The nursing staff mix in my work setting is appropriate		0.71	
4. There are not enough support staff in my work setting[Link]		0.69	
Technology			
1. I do not feel that my access to electronic patient information is adequate[Link]	3.3 (0.8)	0.82	0.75
2. My access to electronic resources (e.g., ordering of tests, access to patient information and results) is adequate for my nursing practice		0.77	
3. I am able to provide better care because of the information systems and technology available to me		0.72	
4. I am not satisfied with the availability of electronic communication with my co‐workers[Link]		0.61	
Autonomy and control			
1. I do not feel that I have an adequate amount of decision‐making freedom in my nursing role[Link]	3.4 (0.7)	0.75	0.74
2. Often I feel like I am not allowed to use my professional nursing judgement to act in my patient's best interest[Link]		0.74	
3. I do not feel that I have direct influence on shaping work environment and how care is provided in my work setting[Link]		0.73	
4. My job description is flexible (I am able to modify my daily duties or the type of work that I do)		0.55	

Note

Scoring: 1 = strongly disagree; 2 = disagree; 3 = neutral; 4 = agree; 5 = strongly agree (including a “not applicable” response option is not recommended). JRIN 6‐factor structure explaining 63% of the variance. Total Cronbach's alpha across all JRIN factors National Survey *α* = 0.88. Case mean imputation guidelines: For each 4‐item subscale, the case mean may be imputed where 25% or less of items is missing (i.e., one item; El‐Masri & Fox‐Wasylyshyn, 2005); if the participant's subscale is missing 2 or more items, then that participant's subscale should be discarded.

Reverse‐scored items: (4, 5, 7, 8, 11, 12, 14, 16, 17, 20, 21, 22, 23).

Table 7 outlines the final subscale dimensions, factor loadings, overall mean subscale scores and reliability for the JDIN scale. Again, factorability was confirmed for the 24 items related to job demands in nursing with Bartlett's Test of Sphericity (*χ* ^2^ = 19,027.17, *df* = 276, *p* < 0.0001) and an acceptable Kaiser‐Meyer‐Olkin measure of sampling adequacy (KMO) of 0.844. An exploratory factor analysis on the items related to job demands originally clustered into six factors with three to four items per factor and a seventh two‐item factor. Due to a low eigenvalue of 1.02, a changing Scree plot slope between the sixth and seventh factor, moderate to low correlation between the two items (*r* = 0.48) and minimal contribution to the total variance, the seventh factor was removed from the model. The factor loadings for the remaining 22 items were ≥0.40, ranging from 0.40‐0.89 (no cross‐loadings), with eigenvalues >1.0 (from 1.28 to 5.67), explaining 59% of the variance. The subscale dimensions based on factor loadings for the JDIN scale were (a) work‐related travel, (b) preparedness/scope of practice (renamed from preparedness/extended scope of practice), (c) equipment and supplies, (d) safety, (e) comfort with working conditions (renamed from workload) and (f) isolation, with scores ranging from 1 (strongly disagree) to 5 (strongly agree). The mean score on each dimension of the JDIN ranged from 1.9‐2.5, with the highest perceived job demands related to Safety (mean: 2.5, *SD*: 0.8) and the lowest job demands related to preparedness/scope of practice (mean: 1.9, *SD*: 0.5). Adequate internal consistency reliability was noted for the JDIN subscales (*α* = 0.56–0.85) and for the full 22‐item JDIN scale (*α* = 0.84) for the national sample analysis.

**Table 7:** Factor structure, reliability and mean scores of the final 22‐item Job Demands in Nursing (JDIN) Scale (*N* = 2,341)

Job demands factors	Mean (*SD*)	Factor loadings	Cronbach's *α*
Work‐related travel			
1. Travelling for my work is tiring	2.4 (0.9)	0.86	0.85
2. I am satisfied with the amount of travel involved in my work[Link]		0.82	
3. I feel that my health has suffered because of my work‐related travel		0.80	
4. Travelling for my work does not interfere with my family life[Link]		0.79	
Preparedness/Scope of practice			
1. I feel that I have the necessary skills to do my work[Link]	1.9 (0.5)	0.87	0.83
2. I feel that I have the necessary knowledge to do my work[Link]		0.86	
3. I do not feel adequately prepared for my area of practice		0.66	
4. I know how to use the necessary equipment for patient care[Link]		0.58	
Equipment and supplies			
1. The equipment that I need to do my work is readily available[Link]	2.4 (0.8)	0.89	0.83
2. The supplies that I require for my work are at‐hand when I need them[Link]		0.88	
3. The equipment needed for patient care is poorly maintained		0.67	
Safety			
1. Working in a rural/remote setting does not pose a personal safety risk for me[Link]	2.5 (0.8)	0.77	0.71
2. There are times that I feel that my personal safety is at risk when I am off‐duty		0.70	
3. There are times that I feel that my personal safety is at risk in my workplace		0.70	
3. My workplace responds appropriately to staff's safety concerns[Link]		0.53	
Comfort with working conditions			
1. I am comfortable with the amount of physical effort required for my work[Link]	2.3 (0.6)	0.76	0.64
2. I am comfortable with the amount of mental effort required for my work[Link]		0.68	
3. I am comfortable working alone[Link]		0.57	
4. I am satisfied with my day‐to‐day routine[Link]		0.44	
Isolation			
1. I have colleagues I can call on for back‐up[Link]	2.3 (0.7)	0.77	0.56
2. I feel a sense of isolation from my colleagues		0.67	
3. I have opportunities to debrief with others after difficult experiences[Link]		0.62	

Note

Scoring: 1 = strongly disagree; 2 = disagree; 3 = neutral; 4 = agree; 5 = strongly agree (including a “not applicable” response option is not recommended). JDIN 6‐factor structure explaining 59% of the variance. Total Cronbach's alpha across all JDIN factors National Survey *α* = 0.84. Case mean imputation guidelines: For each 4‐item subscale, the case mean may be imputed where 25% or less of items is missing (i.e., one item; El‐Masri & Fox‐Wasylyshyn, 2005); if the participant's subscale is missing 2 or more items, then that participant's subscale should be discarded. Case mean imputation should not be performed on the 3‐item subscales; if a participant is missing 1 or more items on the 3‐item subscales, then that participant's subscale should be discarded.

Reverse‐scored items: (2, 4, 5, 6, 8, 9, 10, 12, 15, 16, 17, 18, 19, 20, 22).

### 5.5 Summated scores and relationships to other variables

The mean total score for the final 24‐item JRIN scale was 79.6 (*SD*: 13.1) with a range in scores from 27 to 120 for the full national survey sample indicating a medium to high level of perceived work‐related resources. The perceived work‐related demands for the full national sample were low to medium with a mean total score of 51.1 (*SD*: 9.9) for the 22‐item JDIN scale, range in scores from 22‐99. Pearson's product moment correlations among other concepts measured in the national survey and the JRIN and JDIN are presented in Table 8. The JRIN scale and JDIN scale scores correlated as predicted with weak to moderate significant (*p* < 0.001) correlations with work engagement, burnout, organizational commitment and job satisfaction. Participants with higher scores on the JRIN scale tended to exhibit higher work engagement (*r = *0.37), higher organizational commitment (*r* = 0.28), higher job satisfaction (*r* = 0.51) and lower burnout (*r =* −0.46). Similarly, those participants with higher scores on the JDIN scale tended to exhibit higher levels of burnout (*r = *0.43), lower job satisfaction (*r *= −0.45), lower organizational commitment (*r* = −0.23) and lower work engagement (*r *= −0.34).

**Table 8:** Pearson's product moment correlations of JRIN and JDIN with other variables

	JRIN	JDIN
Work engagement	0.37*	−0.34*
Organizational commitment	0.28*	−0.23*
Job satisfaction	0.51*	−0.45*
Burnout	−0.46*	0.43*

Note

*N* ranged from 2,080 to 2,774 for correlation analyses.

a *p < *0.001.

## 6 DISCUSSION

We have developed two new scales integrating several aspects of nurses’ occupational roles to measure the *global* job demands and job resources of nurses (i.e., applicable to a broad range of nursing designations and practice settings). The Job Resources in Nursing (JRIN) Scale and Job Demands in Nursing (JDIN) Scale were pilot‐tested in a sample of nurses who had current or previous experience practicing in rural and remote settings, with reliability testing and item analyses used to reduce each original scale to six 4‐item subscales (total of 24 items per scale). The new scales were further tested using exploratory factor analysis in a large‐scale national survey of nurses from a variety of designations (RNs, LPNs, RPNs and NPs) across Canada. When tested in a representative sample nationally, good internal consistency reliability was demonstrated for both the JRIN Scale (24‐item, four items per subscale, *α* = 0.88) and the JDIN Scale (22‐item, three to four items per subscale, *α* = 0.84). Exploratory factor analysis of the final 24 items related to job resources favoured a 6‐factor structure, accounting for 63% of the variance, with a final 22 items related to job demands also favouring a 6‐factor structure and accounting for 59% of the variance.

With a possible range of 24–120, the summated scores for the JRIN Scale can be interpreted as low (24–56), medium (57–88) and high (89–120) overall job‐related resources. With fewer total items, the possible range in scores for the JDIN Scale is 22–110, with summated scores interpreted as low (22–51), medium (52–80) and high (81–110) overall job‐related demands. For the national sample, it was evident that they perceived themselves to have relatively low to medium job demands and medium to high job resources related to their work. We were encouraged to find that the total scores on the JRIN were positively correlated with work engagement and organizational commitment and inversely correlated with burnout. Further, the total scores on the JDIN were inversely correlated with organizational commitment and work engagement and positively correlated with burnout, suggesting the potential use of these scales to further explore these occupational outcomes. Although comparisons between nursing groups or predicting occupational outcomes were not the purpose of this analysis, the value of these scales is that they may assist in identifying some of the multidimensional resource gaps and demand pressures that require priority attention across a wide variety of settings. For instance, a review of job demands among remote area nurses concluded that the literature in this field was scarce and that further empirical studies would give health service planners much‐needed information for policy purposes (Lenthall et al., 2009).

It is evident that due to budgetary constraints, higher patient acuity, understaffing and increased workloads, many areas of nursing practice are facing higher demands (Montgomery, Spânu, Băban, & Panagopoulo, 2015). Exposure to chronic work strain is especially concerning for the nursing profession, having negative consequences on both their physiological health and psychological well‐being (Aiken et al., 2013). Both the JRIN and JDIN scales could be used in a variety of ways to assist managers and researchers to better understand some of the factors that may positively or negatively influence the physiological or psychological well‐being of nurses in practice and to identify specific areas to target in terms of developing interventions to increase resources and reduce demands in organizations. An assumption of the job demand‐resource model is that strategies that increase perceived resources actually have a protective effect on employee's occupational well‐being, even in the context of demanding working conditions (Bakker et al., 2005). This is important to note, as it may not always be feasible to directly intervene in reducing overall job demands in nursing practice and it may be helpful to explore whether the presence of higher resources (total summated score on the JRIN) has a protective effect on nurses, even in the face of higher demands (total summated score on JDIN). The scales could also be used to identify and compare specific gaps in resources and/or areas where demands are at their highest. For example, by calculating and comparing the summated mean item score for each of the six subscales in both scales (i.e., summated mean item scores divided by the total number of items), which accounts for the differences in the number of total items (e.g., 3–4) for each subscale and allows for standard comparison across subscales to be made. Comparisons indicating a low degree of agreement (1.0–3.0) to a high degree of agreement (> 3.0) on that particular factor or subscale for either the JRIN Scale or JDIN Scale are therefore straightforward to perform.

To support nurses better in their roles and reduce attrition or nursing turnover, the mean subscale scores could be also used to explore the predictive effect that specific job resources (e.g., staffing, collegial support and technology) and job demands (e.g., safety and working conditions) have on important occupational outcomes such as work engagement, burnout and job satisfaction. As well, the total scores for both scales could be used in more complex multivariate analyses, where models of work engagement and burnout would be explored as potential mediators between higher demands/lower resources and other key outcomes such as psychological health status and organizational commitment (Boudrias et al., 2011).

The lowest job resources mean subscale score found in our analysis was for “Staffing and Time,” indicating that nurse participants had a lower level of agreement about having an appropriate mix of support staff, or adequate time to provide comprehensive care, an area of concern commonly identified across the nursing literature, both rural and urban (Shamian, Kerr, Laschinger, & Thomas, 2002; Twigg, Cramer, & Pugh, 2016). When exploring demands in our analysis, “Safety” demonstrated the highest level of agreement for the participants, indicating that their greatest demand was related to their safety being at risk both in their workplace and when off‐duty. The rising rates of physical violence and verbal abuse against nurses and other healthcare workers (Phillips, 2016) highlight how crucial it is to include measures of “safety” in exploring current demands faced by the nursing profession. We believe that targeting policy change to nursing‐specific areas of concern identified through use of the JRIN and JDIN could assist in developing strategies to address unsafe and/or difficult working conditions (e.g., safety, supervision, isolation and preparedness) and ultimately improve the quality of care provided in rural and urban practice settings.

Historically, nursing workforce studies have focused on homogeneous samples of urban nurses to access larger sample sizes (Molinari & Monserud, 2008). Unique to this study is that the development, refinement and modification of the JRIN Scale and JDIN Scale evolved throughout a three‐phase process over 3 years and the concepts that were retained (e.g., collegial support, staffing and time and comfort with working conditions) were determined to have applicability across both rural and urban nursing practice settings. Further evidence for the validity of the scales is necessary in samples of urban nurses, with a key priority to replicate the study using a strictly urban sample of nurses and to examine the reliability of both scales in large tertiary hospitals and/or primary care settings. Ongoing assessment of JRIN and JDIN would also include exploring the international relevance of these scales in countries with similar diversity as Canada in rural and urban practice such as Australia, New Zealand and the USA. In these geographic contexts, similar issues have been identified including being under‐resourced (Lenthall et al., 2009) and having lower numbers of nurses practicing in sparsely populated geographical locations (Glasser, Peters, & McDowell, 2006). Finally, identifying the occupational roles of nurses across geographic contexts and comparing the experiences of those in strictly urban practice settings with those in a diversity of rural settings will further strengthen our understanding of key occupational outcomes (e.g., work engagement, burnout and occupational commitment) predicted by high demands and low resources in nursing practice while also assisting with health human resource planning.

### 6.1 Limitations

In the pilot survey phase of this study, the authors acknowledge that the sample size of 89 respondents was slightly smaller than the target sample of 100. Instead of calculating a content validity index, the decisions to retain/revise items and subscales involved a content evaluation process (i.e., 16‐member research team and 19‐member advisory team) based on theoretical analysis, item analysis, reliability testing and exploratory factor analysis. The authors also recognize the limitation of developing new instruments in a large national survey while attending to the overlap of related constructs (e.g., removal of the Scheduling subscale). Our inclusion of a “not applicable” category on the items in each scale may have exaggerated the number of missing cases in both factor analyses (i.e., our application of listwise deletion criteria which excluded any cases with at least one missing item in each scale). Between 5% and 10% of participants responded “not applicable” on four items of the job resources technology subscale and 18%–20% on four items of the job demands travel subscale. Case mean imputation for the “not applicable” responses was not calculated due to the potential for artificially high correlations (Tabachnick & Fidell, 2013). Finally, we acknowledge we could have taken a more broad approach to developing scales focused on the resources and demands for healthcare professionals as a whole (e.g., focusing on a diversity of disciplines), but we have chosen to develop tools specifically for assessing the resources and demands for regulated nurses.

## 7 CONCLUSION

The 24‐item Job Resources in Nursing (JRIN) Scale and 22‐item Job Demands in Nursing (JDIN) Scale offer researchers short, simple to administer instruments with acceptable factor structures and good internal consistency reliability as tested in a nationwide cross‐sectional survey. Although developed in the context of rural/remote nursing practice, these scales examine specific job demands and resources that a majority of nurses may experience and may mediate or moderate the impact on specific outcomes (e.g., work engagement, organizational commitment and burnout). Use of these scales could assist researchers and managers to better understand the perceived safety and working conditions of regulated nurses and identify specific resources and demands that require action to improve occupational outcomes. Given that our sample is representative of the population of rural and remote nurses throughout Canada, these scales would allow for comparisons to be made across nursing designations, practice settings and geographical areas. Further psychometric testing with urban samples and across different countries to explore their international relevance and make comparisons among various rural/remote and urban practice settings is necessary.

## CONFLICT OF INTEREST

No conflict of interest has been declared by any of the authors. Patient consent: not applicable in this study.

## ETHICS STATEMNET

The overall project adhered to the ethical principles outlined in the Tri‐Council Policy Statement on Ethical Conduct for Research Involving Humans (TCPS, 2014).

## AUTHOR CONTRIBUTIONS

KLP, JGK, NJS, MLPM, JCK, CPK and KK: pilot study and national study conceptualization and design, national study data collection, interpretation of the pilot study and national study results and manuscript preparation. KLP, JGK, NJS and CPK: analyses of pilot study and national study data. All authors have agreed the final version.

## Appendix 1

**Table A1:** 24‐Item Job Resources in Nursing (JRIN) Scale

1.	I receive adequate praise and recognition from my supervisor for the work that I do
2.	I feel validated by my supervisor for a job well done
3.	My supervisor is concerned with my welfare and the welfare of my colleagues working in this setting
4.	The feedback from my supervisor about the work that I do is not adequate
5.	I do not have enough opportunities for professional growth and development
6.	I am able to access an adequate number of in‐services or continuing education activities
7.	I do not receive adequate training on new technology
8.	I do not feel that a sufficient amount of training is provided when I take on new nursing responsibilities and/or competencies
9.	There is a sense of mutual trust and respect between my colleagues and myself
10.	I feel supported by my colleagues for the work that I do as a nurse
11.	I do not feel that I can rely on my colleagues to help me when I am having a difficult time at work
12.	I do not feel like my colleagues are competent in their roles
13.	There are enough staff members in my work setting to get the job done
14.	I do not have adequate time to do what is important (beyond just basic care) in my nursing role
15.	The nursing staff mix in my work setting is appropriate
16.	There are not enough support staff in my work setting
17.	I do not feel that my access to electronic patient information is adequate
18.	My access to electronic resources (e.g., ordering of tests, access to patient information and results) is adequate for my nursing practice
19.	I am able to provide better care because of the information systems and technology available to me
20.	I am not satisfied with the availability of electronic communication with my co‐workers
21.	I do not feel that I have an adequate amount of decision‐making freedom in my nursing role
22.	Often I feel like I am not allowed to use my professional nursing judgement to act in my patient's best interest
23.	I do not feel that I have direct influence on shaping work environment and how care is provided in my work setting
24.	My job description is flexible (I am able to modify my daily duties or the type of work that I do)

Note

Scoring: 1 = strongly disagree; 2 = disagree; 3 = neutral; 4 = agree; 5 = strongly agree (including a “not applicable” response option is not recommended). Reverse‐scored items: (4, 5, 7, 8, 11, 12, 14, 16, 17, 20, 21, 22, 23). Subscales: supervision, recognition and feedback (1, 2, 3, 4); training, professional development and continuing education (5, 6, 7, 8); collegial support (9, 10, 11, 12); staffing and time (13, 14, 15, 16); technology (17, 18, 19, 20); and autonomy and control (21, 22, 23, 24). Case mean imputation guidelines: For each 4‐item subscale, the case mean may be imputed where 25% or less of items is missing (i.e., one item; El‐Masri & Fox‐Wasylyshyn, 2005); if the participant's subscale is missing 2 or more items, then that participant's subscale should be discarded.

**Table A2:** 22‐Item Job Demands in Nursing (JDIN) Scale

1.	Travelling for my work is tiring
2.	I am satisfied with the amount of travel involved in my work
3.	I feel that my health has suffered because of my work‐related travel
4.	Travelling for my work does not interfere with my family life
5.	I feel that I have the necessary skills to do my work
6.	I feel that I have the necessary knowledge to do my work
7.	I do not feel adequately prepared for my area of practice
8.	I know how to use the necessary equipment for patient care
9.	The equipment that I need to do my work is readily available
10.	The supplies that I require for my work are at‐hand when I need them
11.	The equipment needed for patient care is poorly maintained
12.	Working in a rural/remote setting does not pose a personal safety risk for me
13.	There are times that I feel that my personal safety is at risk when I am off‐duty
14.	There are times that I feel that my personal safety is at risk in my workplace
15.	My workplace responds appropriately to staff's safety concerns
16.	I am comfortable with the amount of physical effort required for my work
17.	I am comfortable with the amount of mental effort required for my work
18.	I am comfortable working alone
19.	I am satisfied with my day‐to‐day routine
20.	I have colleagues I can call on for back‐up
21.	I feel a sense of isolation from my colleagues
22.	I have opportunities to debrief with others after difficult experiences

Note

Scoring: 1 = strongly disagree; 2 = disagree; 3 = neutral; 4 = agree; 5 = strongly agree (including a “not applicable” response option is not recommended). Reverse‐scored items: (2, 4, 5, 6, 8, 9, 10, 12, 15, 16, 17, 18, 19, 20, 22). Subscales: work‐related travel (1, 2, 3, 4); preparedness/scope of practice (5, 6, 7, 8); equipment and supplies (9, 10, 11); safety (12, 13, 14, 15); comfort with working conditions (16, 17, 18, 19); and isolation (20, 21, 22). Case mean imputation guidelines: For each 4‐item subscale, the case mean may be imputed where 25% or less of items is missing (i.e., one item; El‐Masri & Fox‐Wasylyshyn, 2005); if the participant's subscale is missing 2 or more items, then that participant's subscale should be discarded. Case mean imputation should not be performed in the 3‐item subscales; if a participant is missing 1 or more items on the 3‐item subscales, then that participant's subscale should be discarded.

## References

[nop2215-bib-0001] Aiken, L. H. , Clarke, S. P. , Sloane, D. M. , Sochalski, J. A. , Busse, R. , & Clarke, H. (2013). Nurses’ reports on hospital care in five countries. Health Affairs, 20, 43–53. 10.1377/hlthaff.20.3.43 11585181

[nop2215-bib-0002] Aiken, L. H. , & Patrician, P. A. (2000). Measuring organization traits of hospitals: Revised nursing work index. Nursing Research, 49(3), 146–153. 10.1097/00006199-200005000-00006 10882319

[nop2215-bib-0003] Alarcon, G. M. (2011). A meta‐analysis of burnout with job demands, resources and attitudes. Journal of Vocational Behaviour, 79, 549–562. 10.1016/j.jvb.2011.03.007

[nop2215-bib-0004] Al‐Homayan, A. M. , Shamsudin, F. M. , Subramaniam, C. , & Islam, R. (2013). Impacts of job demands on nurses’ performance working in public hospitals. American Journal of Applied Sciences, 10(9), 1050–1060. 10.3844/ajassp.2013.1050.1060

[nop2215-bib-0005] Bakker, A. B. , & Demerouti, E. (2007). The Job demands‐resources model: State of the art. Journal of Managerial Psychology, 22, 309–328. 10.1108/02683940710733115

[nop2215-bib-0006] Bakker, A. B. , Demerouti, E. , De Boer, E. , & Schaufeli, W. B. (2003). Job demands and job resources as predictors of absence duration and frequency. Journal of Vocational Behavior, 62, 341–356. 10.1016/S0001-8791(02)00030-1

[nop2215-bib-0007] Bakker, A. B. , Demerouti, E. , & Euwema, M. C. (2005). Job resources buffer the impact of job demands on burnout. Journal of Occupational Health Psychology, 10, 170–180. 10.1037/1076-8998.10.2.170 15826226

[nop2215-bib-0008] Bakker, A. B. , Demerouti, E. , & Schaufeli, W. B. (2003). Dual processes at work in a call centre: An application of the job demands‐resources model. European Journal of Work and Organizational Psychology, 12, 393–417. 10.1080/13594320344000165

[nop2215-bib-0009] Bakker, A. B. , Hakanen, J. J. , Demerouti, E. , & Xanthopoulou, D. (2007). Job resources boost work engagement, particularly when job demands are high. Journal of Educational Psychology, 99(2), 274–284. 10.1037/0022-0663.99.2.274

[nop2215-bib-0010] Bonett, D. G. (2002). Sample size requirements for testing and estimating coefficient alpha. Journal of Educational and Behavioral Statistics, 27(4), 335–340. 10.3102/10769986027004335

[nop2215-bib-0011] Boudrias, J. S. , Desrumaux, P. , Gaudreau, P. , Nelson, K. , Brunet, L. , & Savoie, A. (2011). Modeling the experience of psychological health at work: The role of personal resources, social‐organizational resources and job demands. International Journal of Stress Management, 18(4), 372–395. 10.1037/a0025353

[nop2215-bib-0012] Carter, M. R. , & Tourangeau, A. E. (2012). Staying in nursing: What factors determine whether nurses intend to remain employed? Journal of Advanced Nursing, 68(7), 1589–1600. 10.1111/j.1365-2648.2012.05973.x 22458811

[nop2215-bib-0013] Comrey, A. L. , & Lee, H. B. (1992). A first course in factor analysis (2nd ed.). Hillsdale, NJ: Lawrence Erlbaum Associates.

[nop2215-bib-0014] Cook, D. A. , & Beckman, T. J. (2006). Current concepts in validity and reliability for psychometric instruments: Theory and application. American Journal of Medicine, 119, 166e7–e16. 10.1016/j.amjmed.2005.10.036 16443422

[nop2215-bib-0015] Crowden, A. (2010). Virtue ethics and rural professional healthcare role. Rural Society, 20, 64–75. 10.5172/rsj.20.1.64

[nop2215-bib-0016] Delobelle, P. , Rawlinson, J. , Ntuli, S. , Malatsi, I. , Decock, R. , & Depoorter, A. M. (2011). Job satisfaction and turnover intent of primary healthcare nurses in rural South Africa: A questionnaire survey. Journal of Advanced Nursing, 67(2), 371–383. 10.1111/j.1365-2648.2010.05496.x 21044134

[nop2215-bib-0017] Demerouti, E. , & Bakker, A. B. (2011). The job demands‐resources model: Challenges for future research. South African Journal of Industrial Psychology, 37, 1–9. 10.4102/sajip.v37i2.974

[nop2215-bib-0018] Demerouti, E. , Bakker, A. B. , Nachreiner, F. , & Schaufeli, W. B. (2000). A model of burnout and life satisfaction among nurses. Journal of Advanced Nursing, 32, 454–464. 10.1046/j.1365-2648.2000.01496.x 10964195

[nop2215-bib-0019] Demerouti, E. , Bakker, A. B. , Nachreiner, F. , & Schaufeli, W. B. (2001). The job demands‐resources model of burnout. Journal of Applied Psychology, 86, 499–512. 10.1037/0021-9010.86.3.499 11419809

[nop2215-bib-0020] DesRoches, C. M. , Miralles, P. , Buerhaus, P. , Hess, R. , & Donelan, K. (2011). Health information technology in the workplace: Findings from a 2010 national survey of registered nurses. Journal of Nursing Administration, 41(9), 357–364. 10.1097/NNA.0b013e31822a7165 21881441

[nop2215-bib-0021] El‐Masri, M. M. , & Fox‐Wasylyshyn, S. M. (2005). Missing data: An introductory conceptual overview for the novice researcher. Canadian Journal of Nursing Research, 37(4), 156–171. 16541824

[nop2215-bib-0022] Furr, R. , & Bacharach, V. (2008). Psychometrics: An introduction. Thousand Oaks, CA: Sage Publications Inc.

[nop2215-bib-0023] Glasser, M. , Peters, K. , & McDowell, M. (2006). Rural Illinois hospital chief executive officers' perceptions of provider shortages and issues in rural recruitment and retention. Journal of Rural Health, 22(1), 59–62. 10.1111/j.1748-0361.2006.00007.x 16441337

[nop2215-bib-0024] Hanvey, L. (2005). Nursing practice in Canada: A discussion paper (draft 3). Canadian Nurses Association (pp. 1–33). Retrieved from https://www.carrn.com/files/Rural-Nursing-discussion-paper_Draft3-Sept%2005-1.pdf

[nop2215-bib-0025] Hayes, L. J. , O'Brien‐Pallas, L. , Duffield, C. , Shamian, J. , Buchan, J. , Hughes, F. , … Stone, P. W. (2006). Nursing turnover: A literature review. International Journal of Nursing Studies, 43(2), 237–263. 10.1016/j.ijnurstu.2005.02.007 15878771

[nop2215-bib-0026] Hunsberger, M. , Baumann, A. , Blythe, J. , & Crea, M. (2009). Sustaining the rural workforce: Nursing perspectives on worklife challenges. Journal of Rural Health, 25(1), 17–25. 10.1111/j.1748-0361.2009.00194.x 19166557

[nop2215-bib-0027] Islam, R. , & Al‐Homayan, A. M. (2013). Impacts of job resources on nurses’ performance working in public sector hospitals. American Journal of Applied Sciences, 10(10), 1224–1233. 10.3844/ajassp.2013.1115.1123

[nop2215-bib-0028] Jourdain, G. , & Chênevert, D. (2010). Job demands–resources, burnout and intention to leave the nursing profession: A questionnaire survey. International Journal of Nursing Studies, 47(6), 709–722. 10.1016/j.ijnurstu.2009.11.007 20138278

[nop2215-bib-0029] Kellar, S. , & Kelvin, E. (2013). Statistical methods for health care research. New York, NY: Wolter Kluwer Health|Lippincott Williams & Wilkins.

[nop2215-bib-0030] Kosteniuk, J. G. , Stewart, N. J. , Karunanayake, C. P. , Wilson, E. C. , Penz, K. L. , Kulig, J. C. , … MacLeod, M. L. P. (2017). Exploratory factor analysis and reliability of the Primary Health Care Engagement (PHCE) Scale in rural and remote nurses: Findings from a national survey. Primary Health Care Research and Development, 18(6), 608–622. 10.1017/S146342361700038X 28747238

[nop2215-bib-0031] Kosteniuk, J. G. , Wilson, E. C. , Penz, K. L. , MacLeod, M. L. , Stewart, N. J. , Kulig, J. C. , … Kilpatrick, K. (2016). Development and psychometric evaluation of the Primary Health Care Engagement (PHCE) Scale: A pilot survey of rural and remote nurses. Primary Health Care Research and Development, 17(1), 72–86. 10.1017/S1463423615000158 25786643

[nop2215-bib-0032] Lavoie‐Tremblay, M. , Trepanier, S. G. , Ferner, C. , & Bonneville‐Roussy, A. (2013). Testing and extending the triple match principle in the nursing profession: A generational perspective on job demands, job resources and strain at work. Journal of Advanced Nursing, 70(2), 310–322. 10.1111/jan.12188 23772766

[nop2215-bib-0033] Lenthall, S. , Wakerman, J. , Opie, T. , Dollard, M. , Dunn, S. , Knight, S. , … MacLeod, M. (2011). Back from the edge: reducing and preventing occupational stress in the remote area nursing workforce. Proceedings of the 11th National Rural Health Conference, Perth WA, March 13–16, 2011. Canberra, ACT: National Rural Health Alliance.

[nop2215-bib-0034] Lenthall, S. , Wakerman, J. , Dollard, M. , Dunn, S. , Knight, S. , Opie, T. , … MacLeod, M. (2018). Reducing occupational stress among registered nurses in very remote Australia: A participatory action research approach. Collegian, 25(2), 181–191. 10.1016/j.colegn.2017.04.007

[nop2215-bib-0035] Lenthall, S. , Wakerman, J. , Opie, T. , Dollard, M. , Dunn, S. , Knight, S. , … Watson, C. (2009). What stresses remote area nurses? Current knowledge and future action. Australian Journal of Rural Health, 17, 208–213. 10.1111/j.1440-1584.2009.01073.x 19664086

[nop2215-bib-0036] Llorens, S. , Bakker, A. B. , Schaufeli, W. , & Salanova, M. (2006). Testing the robustness of the job demands‐resources model. International Journal of Stress Management, 13(3), 378–391. 10.1037/1072-5245.14.2.224

[nop2215-bib-0037] MacLeod, M. L. P. , Stewart, N. J. , Kulig, J. C. , Anguish, P. , Andrews, M. E. , Banner, D. , … Zimmer, L. (2017). Nurses who work in rural and remote communities in Canada: A national survey. Human Resources for Health, 15(34), 1–11. 10.1186/s12960-017-0209-0 28535773 PMC5442670

[nop2215-bib-0038] Mauno, S. , Kinnunen, U. , & Ruokolainen, M. (2007). Job demands and resources as antecedents of work engagement: A longitudinal study. Journal of Vocational Behavior, 70(1), 149–171. 10.1016/j.jvb.2006.09.002

[nop2215-bib-0039] Messick, S. (1989). Validity In Linn R. L. (Ed.), Educational measurement (3rd ed., pp. 13–104). New York, NY: American Council on Education and Macmillan.

[nop2215-bib-0040] Molinari, D. L. , & Monserud, M. A. (2008). Rural nurse job satisfaction. Rural and Remote Health, 1055, 1–12. 19061306

[nop2215-bib-0041] Montgomery, A. , Spânu, F. , Băban, A. , & Panagopoulo, E. (2015). Job demands, burnout and engagement among nurses: A multi‐level analysis of ORCAB data investigating the moderating effect of teamwork. Burnout Research, 2(2–3), 71–79. 10.1016/j.burn.2015.06.001 26877971 PMC4710673

[nop2215-bib-0042] Nahrgang, J. D. , Morgeson, F. P. , & Hofmann, D. A. (2011). Safety at work: A meta‐analytic investigation of the link between job demands, job resources, burnout, engagement, and safety outcomes. Journal of Applied Psychology, 96(1), 71–94. 10.1037/a0021484 21171732

[nop2215-bib-0043] Nelson, W. , Pomerantz, A. , Howard, K. , & Bushy, A. (2007). A proposed rural healthcare ethics agenda. Journal of Medical Ethics, 33, 136–139. 10.1136/jme.2006.015966 17329381 PMC2598268

[nop2215-bib-0044] Nunnally, J. , & Bernstein, I. (1994). Psychometric theory (3rd ed.). New York, NY: McGraw‐Hill Inc.

[nop2215-bib-0045] Orchard, C. , King, G. A. , Khalili, H. , & Bezzina, M. B. (2012). Assessment of interprofesisonal team collaboration scale (AITCS): Development and testing of the instrument. Journal of Continuing Education in the Health Profession, 32(1), 58–67. 10.1002/chp.21123 22447712

[nop2215-bib-0046] Osborne, J. , & Costello, A. B. (2009). Best practices in exploratory factor analysis: Four recommendations for getting the most from your analysis. Pan‐Pacific Management Review, 12(2), 131–146.

[nop2215-bib-0047] Penz, K. , D'Arcy, C. , Stewart, N. , Kosteniuk, J. , Morgan, D. , & Smith, B. (2007). Barriers to participation in continuing education activities among rural and remote nurses. Journal of Continuing Education in Nursing, 38(2), 58–66; quiz 67–8, 93. 17402377 10.3928/00220124-20070301-03

[nop2215-bib-0048] Penz, K. , Stewart, N. , & D’Arcy., & Morgan, D., (2008). Predictors of job satisfaction for rural acute care registered nurses in Canada. Western Journal of Nursing Research, 30(7), 785–800. 10.1177/0193945908319248 18612089

[nop2215-bib-0049] Phillips, J. P. (2016). Workplace violence against health care workers in the United States. New England Journal of Medicine, 374, 1661–1669. 10.1056/NEJMra1501998 27119238

[nop2215-bib-0050] Rudman, A. , Gustavsson, P. , & Hultell, D. (2014). A prospective study of nurses' intentions to leave the profession during their first five years of practice in Sweden. International Journal of Nursing Studies, 51(4), 612–624. 10.1016/j.ijnurstu.2013.09.012 24207027

[nop2215-bib-0051] Schaufeli, W. B. , & Bakker, A. B. (2004). Job demands, job resources and their relationship with burnout and engagement: A multi‐sample study. Journal of Organizational Behavior, 25(3), 293–315. 10.1002/job.248

[nop2215-bib-0052] Schmidt, K. H. , & Diestel, S. (2013). Job demands and personal resources in their relations to indicators of job strain among nurses for older people. Journal of Advanced Nursing, 69(10), 2185–2195. 10.1111/jan.12082 23317358

[nop2215-bib-0053] Shamian, J. , Kerr, M. S. , Laschinger, H. K. S. , & Thomas, D. (2002). A hospital‐level analysis of the work environment and workforce health indicators for registered nurses in Ontario’s acute‐care hospitals. Canadian Journal of Nursing Research, 33(4), 35–50. 11998196

[nop2215-bib-0054] Spence Laschinger, H. K. , Grau, A. L. , Finegan, J. , & Wilk, P. (2012). Predictors of new graduate nurses' workplace well‐being: Testing the job demands‐resources model. Health Care Management Review, 37(2), 175–186. 10.1097/HMR.0b013e31822aa456 21799432

[nop2215-bib-0055] Stewart, N. J. , D'Arcy, C. , Pitblado, J. R. , Morgan, D. G. , Forbes, D. , Remus, G. , … MacLeod, M. L. (2005). A profile of registered nurses in rural and remote Canada. Canadian Journal of Nursing Research, 37(1), 122–145. 15887769

[nop2215-bib-0056] Tabachnick, B. G. , & Fidell, L. S. (2013). Using multivariate statistics. Boston, MA: Pearson.

[nop2215-bib-0057] Thompson, L. (2004). Rural primary care nurses: Governing (im)mobile selves. Health Sociology Review, 13, 178–186. 10.5172/hesr.13.2.178

[nop2215-bib-0058] Twigg, D. E. , Cramer, J. H. , & Pugh, J. D. (2016). Nurse staffing and workload drivers in small rural hospitals: An imperative for evidence. Online Journal of Rural Nursing and Health Care, 16(1), 97–121.

[nop2215-bib-0059] Van den Tooren, M. , & de Jonge, J. (2008). Managing job stress in nursing: What kind of resources do we need? Journal of Advanced Nursing, 63(1), 75–84. 10.1111/j.1365-2648.2008.04657.x 18522680

[nop2215-bib-0060] Williams, E. , Konrad, T. , Linzer, M. , McMurray, J. , Pathman, D. , Gerrity, M. , … Douglas, J. (1999). Refining the measurement of physician job satisfaction: Results from the physician worklife survey. Medical Care, 37(11), 1140–1154. 10.1097/00005650-199911000-00006 10549616

